# A138 SERRATED POLYPOSIS SYNDROME IN INFLAMMATORY BOWEL DISEASE: A SEQUELAE OF CHRONIC DISEASE ACTIVITY OR NEW WHO PHENOTYPE?

**DOI:** 10.1093/jcag/gwac036.138

**Published:** 2023-03-07

**Authors:** S X Jiang, W Xiong, N Shahidi

**Affiliations:** 1 Medicine; 2 Pathology; 3 Gastroenterology, University of British Columbia, Vancouver, Canada

## Abstract

**Background:**

Serrated polyposis syndrome (SPS) is a growing health concern with up to 1 in 125 universal screening program participants meeting diagnostic criteria. Conversely, SPS in inflammatory bowel disease (IBD) is rarely described in the literature, despite the predisposition for serrated epithelial change and serrated polyps in this population.

**Purpose:**

To describe a case of World Health Organization (WHO) criterion I SPS in a patient with IBD, and discuss the existing literature on this rare occurrence.

**Method:**

Case report and review of the literature.

**Result(s):**

**Case Report**

A 53-year-old female with ulcerative colitis (UC; Phenotype: Left-sided; Duration of disease: 40 years; Medical therapy: sulfasalazine) undergoing regular endoscopic surveillance was recently found to have multiple serrated-class lesions including 6 in the sigmoid colon ranging between 8-30mm in size; this included 3 sessile serrated lesions (SSLs) between 20-30mm removed by piecemeal cold snare resection without complication and 2 residual large SSLs for staged endoscopic resection. No endoscopic disease activity was appreciated (Mayo 0), with histopathology of the sigmoid colon and rectum demonstrating chronic inactive colitis.

**Literature Review**

From 2008 to 2021, there are eleven reported cases of SPS in IBD. Six patients had UC and most were in remission for several decades at the time of SPS diagnosis. Most SPS cases met WHO criteria and fit within described phenotypes. While the case above meets WHO criterion I for SPS, the presence of large distal lesions is atypical, raising the question of whether chronic disease activity contributed to the development of these serrated lesions, given the known predisposition for serrated epithelial change and serrated lesions in patients with colonic IBD.

**Image:**

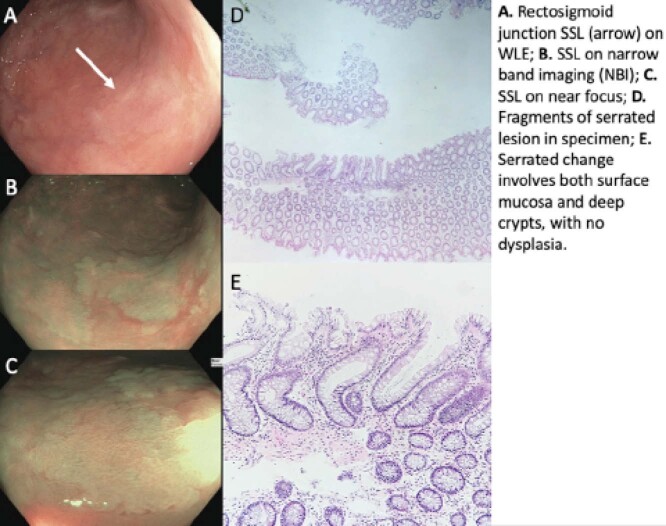

**Conclusion(s):**

SPS in patients with IBD is rare. Questions remain about the role of chronic disease activity contributing to the formation of serrated lesions, its clinical relevance, and optimal management strategy.

**Please acknowledge all funding agencies by checking the applicable boxes below:**

None

**Disclosure of Interest:**

None Declared

